# Supplementary material for: A Random Shuffle Method to Expand a Narrow Dataset and Overcome the Associated Challenges in a Clinical Study: A Heart Failure Cohort Example
Source: Front Cardiovasc Med. 2020 Nov 20;7:599923. doi: 10.3389/fcvm.2020.599923 (PMC7714902; doi:10.3389/fcvm.2020.599923)
Supplement: Supplementary file 1 [file Data_Sheet_2.PDF]

Reproducibility

The codes use the random number generator of MATLAB®, so the results, the Figures 2B and 2C, and the statistics can be different at each script run.

1) Unzip the file Reproducibility.zip to obtain the folder Reproducibility.

2) Open the file stop_sco_aldo_dhf_preprocessed.xlsx. The clinical dataset is composed of a total of 711 German, Austrian and Italian patients suffering from HF in different stages.
In columns A:M, there are the 13 binary (dummy) features: peripheral edema, composite endpoint, age > 75 years, angiotensin receptor blockers intake, β-blockers intake, left ventricular ejection fraction at admission > 50%, nt-proBNP > 1000 pg/mL, diabetes, chronic kidney disease with glomerular filtration rate < 50 mL/min, heart rate at release ≥ 90 bpm, anemia (hemoglobin concentration < 12 g/dL for women, < 13 g/dL for men), all-causes hospitalization endpoint, more than 2 hospitalizations in the last year.
In rows 1:2, columns A:M, there is the calculus for the self-balancing (Knuth 1998) (also called height-balancing) applied to the tree of the binary features; the balancing was used to obtain the sorting of the dataset. In summary, the ordered list of the first 13 columns is the list above.
In columns N:S, there are the 6 numerical features: age, heart rate at release, body weight at release, systolic aortic pressure at release, diastolic aortic pressure at release, left ventricular ejection fraction at admission.
In column T, there is the class label generated via the above-mentioned self-balancing.
All the other columns were not used in this work.
In row 394, the 'Endpoint' exclusion starts: patients were excluded if there is at least an endpoint lacking (composite endpoint, all-causes hospitalization endpoint).
In row 512, the 'Feature' exclusion starts: patients were excluded if there is at least a feature lacking (other than endpoints).
In row 581, the 'Monoesempio' exclusion starts: the mono-example classes (i.e., with a lone patient) were excluded.

3) Start MATLAB® (we used the release R2020a in a Windows 10 PC with 16 GB RAM) and chose the folder Reproducibility as the Current Folder.
The codes with label A produce the two enhanced populations (repeated measure, shuffle) and the related statistics (Hotelling 1931; Trujillo-Ortiz 2020).

In a combined approach, via the codes with label B, the preceding shuffled population can be subjected to a 2× repeated-measure processing and, then, we can perform the related statistics (Hotelling 1931; Trujillo-Ortiz 2020).

4) Codes with label A

Look at line 5 in shuffleA.m, at line 9 in rraA.m, at line 8 in hotellingA.m: there is the number 49 to obtain a final 50× enhancement. You can customize the preceding lines. In our work, we have chosen 133.

Run mainA.m. You will obtain 100 Excel files in circa 15 minutes:

- shuffle_original_data.xlsx: the 385 original patients
- N_shuffled_data.xlsx: (N+1)× shuffle enhancement (the 385 original patients are included)
- rra_original_data.xlsx: the 385 original patients
- N_rra_data.xlsx: (N+1)× repeated measure enhancement (the 385 original patients are included)

Note that shuffle_original_data.xlsx and rra_original_data.xlsx have the same content.

Run hotellingA.m. You will obtain, after circa 4 minutes, the following text in Command Window:

```
Comparison # 1
Y =
   68.9974   70.1766   79.9769  135.2779   79.1377   61.6130
Do you have an expected mean vector? (y/n):
```

In our multivariate problem, we have 6 numerical features and we would enhance the original dataset without generating a different population (p>0.05). So, the original dataset gives the expected multivariate mean vector (EMMV) and, against EMMV=Y, we compare the repeated-measure enhancement *vs*. the shuffle enhancement at a significance level of 0.05.

In other words, for the same enhanced number of patients, we are validating the shuffle enhancement using the repeated-measure enhancement which is an already accepted method: the shuffle enhancement is validated until the p-value is not significant (i.e., the enhanced shuffled population is the same as the original dataset or the enhanced repeated-measure one).

So, answer with y to Do you have an expected mean vector? (y/n):

Then, note the square brackets and answer with [68.9974 70.1766 79.9769 135.2779 79.1377 61.6130] to Give me the expected mean vector:

You will obtain (for example in the present random generation):

P=0.9967 that is…without generating a different population (p>0.05)…

And so on…finally, the workspace is saved.

5) Codes with label B

This analysis must be done after the preceding in 4).

In particular, look at lines 1 and 9 in rraB.m in order to customize i) N_shuffled_data.xlsx (pay attention to the option 'Range' to select all cells with values) and ii) how many times to enlarge N_shuffled_data.xlsx, respectively.

Run mainB.m. In some minutes, you will obtain:

- rra_shuffled_original_data.xlsx: the original shuffled patients
- N_rra_shuffled_data.xlsx: (N+1)× repeated measure enhancement (the original shuffled patients are included)

In hotellingB.m, customize the lines 8 and 9. In particular, N_rra_shuffled_data.xlsx (line 9) is one of the files generated via mainB.m, whereas M_rra_data.xlsx (line 8) is generated via mainA.m. Note that M_rra_data.xlsx is the first file with a higher number of rows than N_rra_shuffled_data.xlsx.

Run hotellingB.m and answer in the same manner as for hotellingA.m…finally, the workspace is saved.

References

Hotelling, H. (1931), "The Generalization of Student's Ratio," *The Annals of Mathematical Statistics*, 2 (3), 360-378. DOI: 10.1214/aoms/1177732979.

Knuth, D. (1998), "Balanced Trees [Section 6.2.3 of Volume 3 (Sorting and Searching)]," in The Art of Computer Programming (Second ed.): Addison-Wesley, ISBN 0-201-89685-0, pp. 458-481.

Trujillo-Ortiz, A. (2020), "HotellingT2," *MATLAB Central File Exchange*, https://www.mathworks.com/matlabcentral/fileexchange/2844-hotellingt2842.
